# Sulfur-Containing Amino Acid Plasma Concentrations in Pregnancy; Effects of Physical Activity Interventions and Associations with Neonatal Outcomes—The FitMum Randomized Controlled Trial

**DOI:** 10.3390/nu18172878

**Published:** 2026-09-02

**Authors:** Ida Karoline Bach Jensen, Caroline Borup Roland, Samuel Addison Jack Trammell, Signe de Place Knudsen, Anne Dsane Jessen, Jane Marie Bendix, Stig Molsted, Trisha Jean Grevengoed, Bente Merete Stallknecht, Ellen Løkkegaard, Ole Hartvig Mortensen

**Affiliations:** 1 Department of Gynecology and Obstetrics, Copenhagen University Hospital-North Zealand, 3400 Hilleroed, Denmark; 2 Department of Biomedical Sciences, University of Copenhagen, 2200 Copenhagen, Denmark; 3 Department of Clinical Research, Copenhagen University Hospital-North Zealand, 3400 Hilleroed, Denmark; 4 Department of Clinical Medicine, University of Copenhagen, 2200 Copenhagen, Denmark

**Keywords:** physical activity, exercise intervention, pregnancy, birth weight, placenta, transport, amino acids, methionine, cysteine, cystine, taurine

## Abstract

**Background:** Sulfur-containing amino acids (SCAAs) are essential for fetal growth and development, but their metabolism during pregnancy remains poorly understood, with conflicting reports regarding the effect of exercise training. This study investigates the effects of physical activity (PA) interventions on maternal plasma concentrations of SCAAs and their associations with neonatal outcomes. **Methods:** The FitMum randomized controlled trial included 220 healthy, inactive, pregnant women enrolled before gestation age (GA) 15+0 weeks. Participants were randomized to supervised exercise training (EXE), motivational counseling on PA (MOT), or standard care (CON). This secondary analysis includes all participants with an eligible blood sample (*n* = 214). Maternal plasma concentrations of SCAAs were measured at four time points (GA ≤15+0, 28+0-6, 34+0-6, and delivery), and umbilical cord venous plasma was measured at delivery. **Results:** Longitudinally, maternal methionine and taurine concentrations decreased significantly (*p* < 0.001 for both) during pregnancy, while total cysteine (tCys) concentrations increased (*p* < 0.001). At GA 28+0-6 weeks, methionine concentrations were significantly higher in EXE (*p* = 0.031) and MOT (*p* = 0.006) groups compared to CON, and tCys was higher in the MOT group (*p* = 0.017) versus CON. Maternal taurine was positively associated with birth weight (*p* = 0.034) and birth weight/placental weight ratio (*p* = 0.010), while maternal tCys was negatively associated with head circumference (*p* < 0.001). Additionally, increased moderate-to-vigorous intensity physical activity (MVPA) was associated with a smaller maternal–umbilical venous difference for methionine (*p* = 0.025), suggesting enhanced placental transport. **Conclusions:** Pregnancy significantly alters maternal metabolism of SCAAs. PA interventions may modulate maternal methionine and tCys concentrations, and our results suggest that maternal taurine status is linked to offspring birth weight, highlighting the potential of PA to influence key nutrients required for fetal development.

## 1. Introduction

Regular physical activity (PA) during pregnancy is associated with improved maternal and fetal health outcomes and a reduced risk of several pregnancy complications [1]. PA during pregnancy is known to favorably influence glucose metabolism [2,3] and may also reduce pregnancy-associated dyslipidemia, although not in all studies [4,5,6]. However, the effect of PA on amino acid (AA) metabolism remains largely unexplored. 

AAs are essential for a healthy pregnancy and normal fetal growth and development [7]. AA metabolism changes during pregnancy to meet increased fetal demands, as demonstrated by decreased nitrogen excretion in pregnant vs. non-pregnant women [8]. However, limited research has examined changes in the metabolism or placental transport of specific AAs during pregnancy.

Traditionally, nine AAs have been described as the essential AAs, with an additional AA, arginine, also being essential in the fetus and neonate due to limited synthesis capacity [9]. However, to some extent, this designation primarily reflects the role of these AAs in protein synthesis, rather than their metabolic functions [10]. The sulfur-containing amino acids (SCAAs) include methionine, cysteine, and taurine. Cysteine exists in a redox-dependent equilibrium with its oxidized dimer, cystine, and the balance between these two forms is determined by the oxidation-reduction potential of the environment. This relationship is biologically critical, as cystine is the primary form transported into cells, where it is subsequently reduced back to cysteine to serve as the rate-limiting precursor for the synthesis of glutathione, the body’s master antioxidant [11].

They are among the most metabolically important AAs as they are not only incorporated into proteins (methionine, cysteine), but are also essential precursor molecules, involved in methylation, anti-oxidant defense, and may also act as mediators affecting metabolism and cell function [12]. Taurine plays a role in several important biological processes, including central nervous system development, membrane stabilization, and mitochondrial biogenesis, and is the main osmolyte in mammals [13]. Furthermore, taurine is involved in cholesterol degradation and lipid absorption from the intestines [14]. Taurine is particularly important for fetal growth and development [15,16] and has been suggested as a marker of fetal well-being [17]. The placenta cannot convert methionine to cysteine [18], but appears capable of converting cysteine to taurine [19]; however, its synthesis capacity remains unknown. Taurine is the most abundant AA in placental tissue, with placental concentrations 100- to 200-fold higher than those found in maternal blood [20], suggesting efficient transport to the placenta, similar to other metabolically active tissues [13]. Adequate dietary protein intake is important for maintaining sufficient plasma concentrations of SCAAs during pregnancy. However, in preeclampsia, maternal plasma cysteine concentrations are elevated, while methionine concentrations are reduced [21]. PA may also affect circulating concentrations of SCAAs, as demonstrated in studies of non-pregnant populations, which showed that an exercise training intervention in women resulted in an increase in serum methionine concentrations, with no change in cysteine or taurine concentrations [22], while another study showed no changes in either SCAA [23]. In men, an exercise training intervention resulted in decreased plasma concentrations of both cysteine and taurine, with methionine unchanged [24]. To our knowledge, no studies have previously investigated the effect of PA during pregnancy on plasma concentrations of SCAAs.

We used data from the FitMum randomized controlled trial [25] to investigate changes in concentrations of SCAAs during pregnancy and the effect of PA on plasma concentrations of methionine, total cysteine, and taurine. We also investigated the effect of PA on the maternal-to-umbilical venous concentration difference of methionine, total cysteine, and taurine. Finally, we investigated the associations between maternal and umbilical cord plasma concentrations of methionine, total cysteine, and taurine during pregnancy with birth weight, birth length, and head circumference, as well as placental weight. 

## 2. Materials and Methods

### 2.1. Participants and Study Design

The FitMum study was a randomized controlled trial conducted at the Department of Obstetrics and Gynecology at Nordsjaellands Hospital in Hilleroed, Denmark, from 2018 to 2021. The study included 220 healthy, inactive, pregnant women. As previously described [25], the inclusion criteria were (1) written informed consent, (2) age ≥ 18 years, (3) ultrasonographically confirmed intrauterine pregnancy, (4) GA ≤ 15+0 weeks, (5) body mass index (BMI) between 18.5 and 45 kg/m^2^, and (6) willingness and ability to wear a wrist-worn activity tracker. Exclusion criteria were (1) regular moderate-to-vigorous intensity physical activity (MVPA) (≥1 h/week) during early pregnancy, (2) previous preterm delivery, (3) pre-existing obstetric or medical complications, (4) multiple pregnancies, (5) inability to speak Danish, or (6) alcohol or drug abuse.

Demographic data (e.g., educational level, marital status, number of children) were collected via a structured interview at the inclusion visit, and participants were equipped with an activity tracker. Pre-pregnancy BMI was calculated from self-reported weight and height. 

Following a one-week baseline period, participants were randomized in a 2:2:1 ratio to either structured, supervised exercise training (EXE), motivational counseling on PA during pregnancy (MOT), or a control group receiving standard care (CON). Participants in EXE were offered 3 exercise sessions per week during pregnancy, with 2 of 3 sessions consisting of 30 min of cardiovascular exercise followed by 30 min of resistance training in a gym, and the last session consisting of 15 min of cardiovascular exercise followed by 45 min of resistance exercise in a swimming pool. Participants in MOT were offered 7 motivational counseling sessions during pregnancy, with personalized plans for increasing their PA level. The primary outcome of the FitMum study was the total minutes per week of moderate-to-vigorous intensity physical activity (MVPA) from randomization to GA week 28. The primary aim was to assess differences in weekly minutes of MVPA among the three study groups. The FitMum study was approved by the Regional Committee on Health Research Ethics (30 August 2018, approval number H-18011067) and the Danish Data Protection Agency (12 September 2018, registration number P-2019-512) and registered at clinicaltrials.gov (NCT03679130).

A detailed description of the trial protocol can be found elsewhere [25], and includes: changes to the trial; randomization allocation, blinding and method; sample size determination; predetermined primary, secondary and additional outcomes; statistical analysis plan, involvement of the public and patients in trial design, conduct and reporting; adverse and serious adverse events; concomitant care. Adherence as well as primary and secondary outcome results can be found elsewhere [26,27,28].

### 2.2. Physical Activity Measurements

PA was continuously monitored from inclusion until delivery using a wrist-worn commercial activity tracker (Garmin vívosport, Garmin International Inc., Olathe, KS, USA) with a built-in accelerometer and heart rate monitor. The tracker recorded heart rate every 15 s and daily hours of sedentary time. Minutes of MVPA were calculated using heart rate data and defined as time with a heart rate corresponding to ≥64% of the theoretical maximum heart rate [29]. The theoretical maximum heart rate was defined as 208 − (0.7 × age). Sedentary time was defined as awake time spent with little or no activity, like sitting or resting. The activity tracker measured total sedentary time, including time spent sleeping, and estimated daily sleep duration. Sleep duration was subtracted from total sedentary time to determine awake sedentary time. 

### 2.3. Maternal and Umbilical Cord Plasma Collection

Non-fasting maternal venous blood samples were collected at GA week 6+1 to 15+0, 28+0-6, 34+0-6, and within one hour after delivery (GA week 32+1 to 42+0). Blood samples were collected in EDTA plasma tubes at all timepoints. At delivery, additional maternal plasma samples were collected in CPD tubes. Maternal plasma samples were centrifuged within a few hours of collection and stored at −80 °C until analysis. 

Venous umbilical cord blood was collected within 30 min of placental delivery using a closed collection system containing 10 mL of CPD (T2950, Fresenius Kabi, Bad Homburg, Germany). Umbilical cord blood with CPD was then diluted with PBS to a 1:1 concentration of dilutant (CPD+PBS) to blood and transferred to a sepmate tube (Stemcell, Vancouver, BC, Canada) with Lymphoprep (Stemcell). If there was less than 10 mL of collected blood, the blood was not further diluted, and the dilution factor was noted. The sepmate tube was centrifuged at 1200× *g* for 10 min and the plasma layer was extracted and stored at −80 °C until analysis. 

Placental weight, which included membranes and umbilical cord, was recorded prior to umbilical cord blood collection. For participants without a cord blood sample, the placental weight was retrieved from clinical medical records. Neonatal outcomes, including birth weight, birth length, and head circumference, were also obtained from clinical medical records. 

### 2.4. Maternal and Umbilical Cord Sulfur-Containing Amino Acid Concentrations

Concentrations of SCAAs were quantified using liquid chromatography-tandem mass spectrometry as described below. Total cysteine (tCys, representing the cysteine + cystine concentration) was measured as cystine, its oxidized form, as no antioxidants were added to the samples.

Umbilical cord plasma concentrations of SCAAs were multiplied by a calculated dilution factor, to determine the concentration in undiluted plasma. Plasma samples were analyzed in two batches, with samples randomly assigned to minimize batch effects. For a subset of maternal plasma samples (*n* = 10), SCAAs were measured in both CPD and EDTA plasma to assess the comparability of the values (Table A1). 

Methionine quantification failed in two umbilical cord plasma samples, while tCys and taurine quantification failed in 11 and 4 samples, respectively. At least one of the SCAAs was successfully quantified in 98 of the umbilical cord plasma samples. 

Hemolysis, indicated by redness, was observed in some umbilical cord plasma samples. Hemolysis can artificially elevate concentrations of SCAAs, particularly taurine, due to its abundance in platelets and granulocytes [30]. To account for this, hemoglobin concentration was measured in all cord plasma samples to assess the degree of hemolysis (Hemoglobin Assay MAK115, Sigma-Aldrich, Merck, Darmstadt, Germany). One sample had a hemoglobin concentration exceeding 6 mmol/L, indicating significant hemolysis, and was therefore excluded from the final analyses, resulting in a total of 97 umbilical cord plasma samples.

### 2.5. Plasma Amino Acid Quantitation via Liquid Chromatography-Mass Spectrometry

All solvents were liquid chromatography-mass spectrometry grade unless otherwise stated. All chemicals used for plasma amino acid quantification were from Sigma-Aldrich unless otherwise stated. Amino acid isotopologoues were from Cambridge Isotope Laboratories. Plasma samples were thawed on wet ice after removal from −80 °C storage. A volume of 2.5 µL of plasma was added to 97.5 µL of −20 °C cooled acetonitrile:methanol:water (2:2:1 (*v*/*v*/*v*)) containing 125 pmol of [^13^C5, ^15^N]-L-methionine, [d4]-taurine, and 62.5 pmol of [^13^C6, ^15^N2]-L-cystine. The extracts were immediately vortexed and incubated at −20 °C for 30 min. Extracts were centrifuged at 16,100× *g* for 10 min. A volume of 80 µL of supernatant was transferred to a fresh autosampler vial (Phenomenex Verex vials, Cat# AR0-3920-13, Phenomenex, Torrance, CA, USA). Volumes of 2 µL of the first and last 75 samples from each extraction set were combined to serve as the quality control (QC) pooled sample. Blank extract was prepared by replacing the volume of plasma with water. Four pooled human plasma samples (Merck, catalog # P9523) from healthy individuals were also extracted to serve as QCs. Samples were analyzed in two batches, each using its own standard curve and standard QCs. Standard QCs and external standards were prepared with the same amount of internal standard as used for samples. Standard QCs were prepared in nine replicates per analytical batch at three abundance levels (1.2, 8, and 40 pmol loaded). External standards were prepared at 12 abundance levels (0.01, 0.05, 0.1, 0.3, 1, 3, 10, 30, 37.5, 50, 150, and 200 pmol loaded). All injectables were capped (Phenomenex Verex caps Cat# AR0-8952-13-M) and placed in a prechilled (6.7 °C) autosampler.

Samples were analyzed in random order. QCs were injected throughout the analysis. The standard curve was analyzed after all samples were injected. Samples were injected (2 µL) into a Waters ACQUITY UPLC I-Class PLUS System and separated using liquid chromatography and detected with a Waters Xevo TQ-XS mass spectrometer operated in multiple-reaction monitoring (MRM) mode as previously described [31,32] with slight modification. Two MRM transitions were added to the analysis: taurine (retention time = 5.65 min, precursor ion [M-H]-: 124.01, product ion [M-H]-: 79.71, cone voltage = 46 V, collision energy = 16 eV) and [d4]-taurine (retention time = 5.62 min, precursor ion [M-H]-: 127.88, product ion [M-H]-: 79.71, cone voltage = 46 V, collision energy = 16 eV). The original standard curve for cystine quantification did not meet the criteria for goodness-of-fit (R^2^ ≥ 0.99) and theoretical error at each standard (≤20%). Therefore, a new standard curve, generated with samples in an unrelated experiment, was used for cystine quantification. QC standards from the original experiment at concentrations of 0.3 and 2 micromolar had theoretical errors of 11 ± 4.6% and 10 ± 4%, respectively, when calculated with the new standard curve. The quantified data were within this concentration range and thus had acceptable measurement error. The replacement standard curve was generated using the exact same instrument, instrument parameters, sample preparation protocol, and matrix as the original sample batches. To validate the use of the replacement standard curve, QC standards processed during the original analytical runs (April/May 2024) were quantified using the replacement curve (August 2024). The theoretical errors for these QCs were 8 ± 3.40% and 14 ± 4.10% for the 0.3 micromolar standard, and 10 ± 3.00% and 9 ± 5.00% for the 2 micromolar standard across two days, demonstrating strong inter-batch stability. The lower limit of quantification for cystine was 0.05 pmol, which is lower than any of the samples measured.

### 2.6. Statistical Analyses

Data are presented as means with standard deviations for normally distributed data, and as medians with interquartile range for skewed data. PA means from the tracker were daily means. Days with more than 6 h of missing activity tracker data were excluded. Missing activity tracker data due to non-wear time were imputed using multiple imputation with random forest models, generating 25 datasets using the mice version 3.18 R packages [33], as previously described [28]. This paper includes secondary outcomes from the FitMum RCT as described in the study protocol [25], and a power calculation was not performed for these outcomes.

Differences in maternal SCAAs between visits were analyzed using linear mixed models, to account for the repeated observations and unequal number of blood samples obtained from each participant. Differences between maternal and umbilical cord blood concentrations of SCAAs were tested with a paired *t*-test for normally distributed data and with Wilcoxon signed-rank test for skewed data. All statistical analyses of umbilical cord plasma were adjusted for hemoglobin concentration. 

Differences in methionine, tCys, and taurine concentrations between and within study groups were analyzed using the constrained linear mixed model of Liang and Zeger [34]. This model was applied because it leverages the randomized study design by constraining baseline group averages to be equal, increasing statistical power while still allowing for efficient estimation of changes over time. Estimated means are presented with 95% Wald confidence intervals. All participants were included, regardless of missing samples. Missing observations were assumed to be missing at random. Analyses were adjusted for batch number. 

The maternal–umbilical venous concentration difference was calculated as maternal plasma concentration minus umbilical cord plasma concentration. Intervention group differences in the maternal–umbilical venous difference were tested using analysis of variance (ANOVA). The ANOVA was adjusted for hemoglobin concentration and analysis batch. 

Associations between minutes of MVPA, as well as hours of sedentary time and changes in concentrations of SCAAs with increasing GA, were explored using linear regression analyses with an interaction term between PA measure (MVPA and sedentary time) and GA (GA:PA measure). The GA:PA interaction term was included to assess whether the association between gestational age (GA) and plasma concentrations of SCAAs was modified by physical activity. This approach allowed us to determine if the longitudinal change in concentrations of SCAAs over the course of pregnancy differed significantly based on the levels of MVPA or sedentary time.

The analyses included plasma concentrations of SCAAs from all time points, and all participants were included regardless of missing samples, as observations were assumed to be missing at random. These analyses were adjusted for maternal age, pre-pregnancy body mass index, and analysis batch. Cluster robust standard errors were applied to account for repeated measurements using the sandwich R package [35,36]. PA measures included in these analyses were daily means at baseline, from randomization to GA week 28+0-6, GA week 28+0-6 to 34+0-6, and GA week 34+0-6 to delivery, respectively.

Associations between minutes of MVPA and hours of sedentary time with the maternal–umbilical venous difference were analyzed using multiple linear regression, adjusting for maternal age, pre-pregnancy body mass index, hemoglobin concentration, and analysis batch. PA measures in these analyses were daily means from randomization to delivery.

Associations between concentrations SCAAs in maternal and umbilical cord plasma and placental weight, birth weight, and birth weight/placental weight ratio (BW/PW) were analyzed with linear regression. To increase statistical power, results from maternal plasma samples at all four time points were included, and cluster robust standard errors using the sandwich R package [37] were applied to account for repeated measurements. Adjusted and unadjusted results are presented for maternal results. In the adjusted models, analyses were adjusted for maternal age, GA, offspring sex, and maternal pre-pregnancy BMI. In addition, analyses with umbilical cord plasma were adjusted for hemoglobin concentration.

All statistical analyses were performed in R version 4.5.0, and statistical significance level was defined as a *p*-value below 0.05.

## 3. Results

### 3.1. Participants 

**Figure 1:**
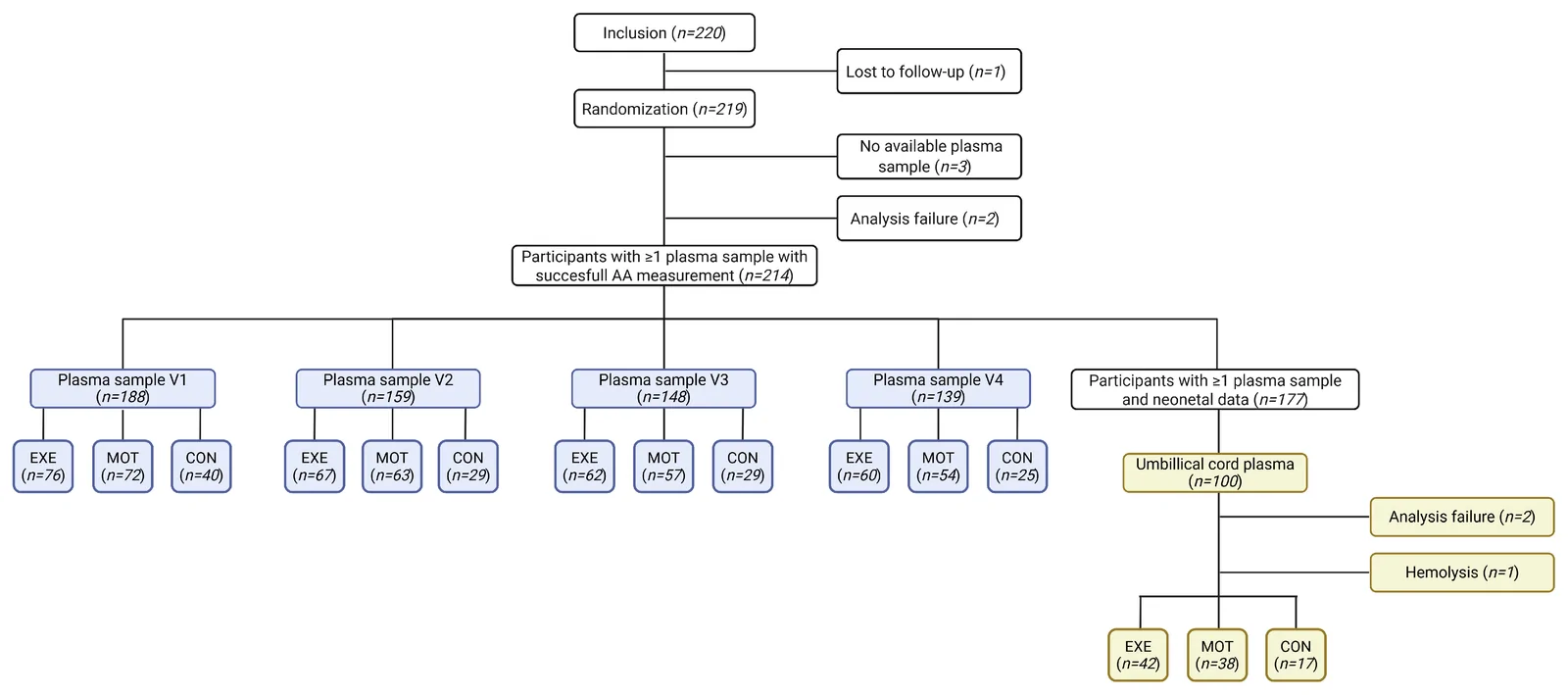
Flow diagram of inclusion, randomization, study group allocation, and maternal and umbilical cord plasma samples. V1, Gestational age ≤15+0 weeks; V2; Gestational age week 28+0-6; V3, Gestational age week 34+0-6; V4, delivery; AA, Amino acid; EXE, Structured supervised exercise training; MOT, Motivational counseling on physical activity; CON, Standard care. Figure created with BioRender.com.

A flowchart of participants with blood samples taken during pregnancy, as well as umbilical cord blood samples, is presented in Figure 1. A total of 216 participants provided at least one plasma sample, and AA concentrations were detected in plasma samples from 214 participants. Maternal and neonatal characteristics are presented in Table 1.

### 3.2. Sulfur-Containing Amino Acid Concentrations in Maternal and Fetal Plasma

Mean maternal and umbilical cord plasma concentrations of methionine, tCys, and taurine from all participants at all time points in pregnancy are presented in Table 2. Overall, methionine concentrations decreased after GA 34+0-6 weeks compared to GA ≤15+0 weeks to a maximum decrease of 10% (−2.2 μmol/L [−3.5; −0.1], *p* < 0.001) from GA ≤15+0 weeks to delivery, while tCys concentrations initially slightly decreased at GA 28+0-6 weeks compared to GA ≤15+0 weeks, but ended up increasing by 27% (9.9 μmol/L [7.6; 12.3], *p* < 0.001) from GA ≤15+0 weeks to delivery (Table 2). Taurine concentrations decreased throughout pregnancy compared to GA ≤15+0, to a maximum decrease of 36% (−23.8 μmol/L [−28.8; −18.7], *p* < 0.001) from GA ≤15+0 weeks to delivery (Table 2). 

At delivery, methionine and tCys concentrations were lower in umbilical cord plasma than in maternal plasma, while taurine concentrations on average were slightly higher in umbilical cord plasma than in maternal plasma (Table 2).

### 3.3. Sulfur-Containing Amino Acids in Intervention Groups

**Figure 2:**
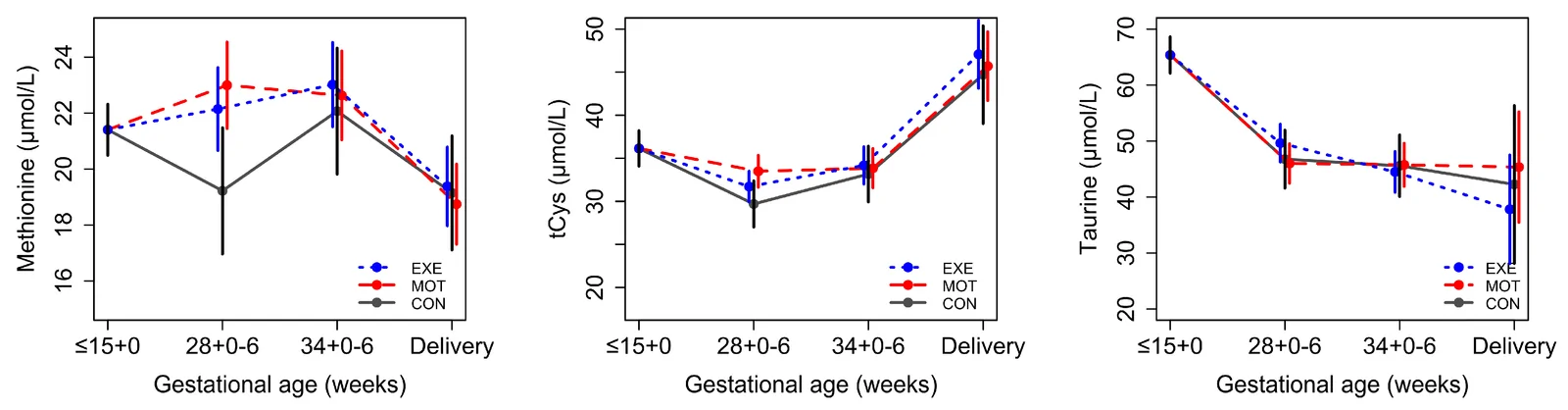
Maternal plasma methionine, total cysteine, and taurine concentrations across pregnancy. Mean maternal plasma methionine, total cysteine, and taurine concentrations with 95% confidence intervals in the three study groups at baseline (gestational age week ≤15+0), gestational age week 28+0-6, 34+0-6, and at delivery (GA week 32+1 to 42+0). A small horizontal jitter was added for visual clarity. tCys, total cysteine; CON, Standard care; EXE, Structured supervised exercise training; MOT, Motivational counseling on physical activity.

Methionine concentrations were 15% higher in participants in the EXE group (*p* = 0.031) and 20% higher in the MOT (*p* = 0.006) group compared to the CON group at GA week 28+0-6 (Table 3 and Figure 2). tCys concentrations were 14% higher in participants in the MOT (*p* = 0.017) group compared to the CON group at GA week 28+0-6. The intervention had no effect on taurine concentrations at any time point during pregnancy. As a measure of placental transport efficiency, we calculated the maternal–umbilical venous difference (MUV-difference), which we defined as the maternal plasma concentration minus the umbilical cord plasma concentration at delivery, but found no significant effect of the interventions on this (Table 4).

### 3.4. Association Between Objective Physical Activity Levels and Sulfur-Containing Amino Acid Concentrations

No significant effect modification by either MVPA or sedentary time on the association between GA and either methionine, tCys, or taurine plasma concentrations was observed (Table 5). This indicates that although taurine and tCys plasma concentrations both change as pregnancy progresses, the rate of this change was not modified by PA level or sedentary time.

Increased minutes of MVPA were associated with a smaller difference between maternal and umbilical cord blood plasma concentrations of methionine (*p* = 0.025) (Table 6). No associations were detected between MVPA and tCys or taurine maternal–umbilical venous difference, and no associations were found between sedentary time and maternal–umbilical venous difference for any of the measured SCAAs (Table 6).

### 3.5. Sulfur-Containing Amino Acids and Neonatal Outcomes

Maternal plasma taurine concentrations were significantly associated with birth weight in adjusted analyses, and with BW/PW in both unadjusted and adjusted models (Table 7). tCys concentration in maternal plasma was also negatively associated with head circumference in both adjusted and unadjusted analyses (Table 7). No associations were detected between umbilical cord plasma concentrations of either methionine, tCys, or taurine and any of the neonatal outcomes.

## 4. Discussion

Our findings demonstrate that maternal metabolism of SCAAs is significantly altered during pregnancy, with a notable lower concentration of methionine and taurine and a higher concentration of tCys at delivery compared to early pregnancy. The observed transient increases in methionine and tCys in the intervention groups suggest that PA may modulate the availability of these critical nutrients in mid-pregnancy. These changes are particularly relevant given the essential roles that SCAAs play in fetal organogenesis [38], placental function [39,40], and the protection of the maternal-fetal unit from oxidative stress [41,42].

### 4.1. Changes in Sulfur-Containing Amino Acid Concentrations During Pregnancy

Studies investigating changes in AA concentrations during pregnancy have yielded inconsistent results. In contrast to our results, one study reported increased methionine concentrations during pregnancy [43], while another study found no significant changes in methionine concentrations from the first to third trimester [44]. The latter findings align with our results, as the decrease observed in our study occurred from GA ≤15+0 weeks to delivery. We did not observe any significant changes when comparing GA week ≤15+0 with week 34+0 (third trimester). The observed decrease in methionine concentrations in our study may be due to increased utilization during late pregnancy or consumption during labor, as plasma samples were collected shortly after delivery. One possibility is that the physiological stress and intense myometrial contractions of labor may mimic the effects of acute exercise, potentially contributing to the observed decrease in methionine, as acute exercise is associated with a decreased methionine plasma concentration [45]. In contrast to our findings, one previous study found no changes in cystine concentrations during pregnancy [43].

We found a significant decrease in taurine plasma concentrations, particularly from GA ≤15+0 to 28+0-6 weeks, suggesting substantial placental transfer or uptake during this period. This aligns with one previous study [43], but is contradicted by another study that found an increase during pregnancy, but a decrease when compared to non-pregnant women [46]. Further research with larger populations is needed to fully elucidate changes in methionine, cysteine, and taurine plasma concentrations during pregnancy.

The dynamics of plasma concentrations of SCAAs during pregnancy are closely linked to the high metabolic demands of the developing fetus. Methionine serves as the precursor for S-adenosylmethionine, the universal methyl donor required for DNA and histone methylation [47]. These epigenetic modifications are critical for regulating fetal gene expression and embryonic development [48]. The observed decrease in maternal methionine toward the end of pregnancy may reflect an increased demand for methylation reactions to support late-gestational fetal growth.

Simultaneously, the increase in tCys concentration observed in our study may reflect the pregnancy-induced demand for antioxidant defense. Cysteine is the rate-limiting precursor for the synthesis of glutathione, the primary intracellular antioxidant [11]. The placenta and fetus are particularly vulnerable to oxidative stress [49], and the upregulation of cysteine availability might be essential to protect fetal tissues from reactive oxygen species that increase during the third trimester [50].

The observed changes in tCys concentrations are of particular interest when considering the redox status of the maternal-fetal unit. Cysteine and cystine exist in a dynamic equilibrium that is highly sensitive to the oxidative environment, which can be modulated by both the physiological stress of late pregnancy [50] and the metabolic demands of PA [51]. A potential shift in this redox balance is biologically significant, as it determines the availability of reduced cysteine for the synthesis of glutathione, the primary intracellular antioxidant required to protect the fetus from oxidative stress.

### 4.2. Physical Activity and Sulfur-Containing Amino Acids 

Methionine plasma concentrations were higher in both the EXE and MOT groups compared to the CON group at GA week 28+0-6. tCys plasma concentrations increased only in the MOT group compared to CON, also at GA week 28+0-6. PA interventions did not significantly alter taurine plasma concentrations. We did not find any correlation between objective measures of PA and plasma concentrations of SCAAs. To our knowledge, no studies have previously investigated the effects of a PA intervention or objectively measured effects of PA on concentrations of SCAAs during pregnancy. Previous exercise training studies in non-pregnant women have reported conflicting findings regarding methionine. One study observed an increase in serum methionine [22], while another found no difference in plasma methionine [23]. Our tCys results contrast with two other studies that reported no increase [22,23]. One study in men reported a decrease in plasma taurine following exercise [24], which contradicts our finding of no effect of PA on taurine concentration. Taurine metabolism is substantially altered during pregnancy [15,16], potentially explaining differences in the effect of exercise on plasma taurine concentrations between pregnant and non-pregnant individuals. Several factors may explain the differing effects of PA on plasma concentrations SCAAs, including variations in training regimes, differences between plasma and serum analysis [22], sex [24], and potential weight loss as a confounding variable [23]. 

This highlights the need for further research on the relationship between exercise and concentrations SCAAs in the circulation in carefully controlled trials where both sex and pregnancy-related effects are considered.

### 4.3. Physical Activity and Maternal–Umbilical Cord Concentration Difference of Sulfur-Containing Amino Acids

Placental transport of AAs remains poorly understood. AAs are actively transported across the placenta [52], and studies have shown that fetal plasma taurine concentrations are higher than maternal concentrations [53], consistent with our findings. We found no effect of the PA interventions on the difference in concentrations between maternal and umbilical cord venous plasma. However, increased minutes of MVPA were associated with a smaller difference in methionine concentration between maternal and umbilical cord venous plasma at delivery. This suggests that increased PA may enhance placental methionine transport. However, the results are exploratory, and future studies investigating the difference between maternal blood concentrations and umbilical cord arterial concentrations are needed to conclude on this.

### 4.4. Associations Between Sulfur-Containing Amino Acid Concentrations and Neonatal Outcomes

We found that an increase in maternal taurine concentration of 1 micromolar was associated with an increase in birth weight of 3.25 g, corresponding to a potential 860 g difference in birth weight between the participant with the lowest taurine concentration at delivery (17.2 micromolar) and the participant with the highest taurine concentration (281.7 micromolar). When comparing the three lowest with the three highest taurine concentrations at delivery, for women giving birth at term, we get an average difference in taurine concentration of 210.2 micromolar and a birth weight difference of 598 g, corresponding to a 2.84 g increase in birth weight per 1 micromolar taurine concentration increase, corroborating this observation. Though the results are exploratory, they emphasize the importance of further research into the effect of taurine on fetal growth and development.

To our knowledge, this is the first study to demonstrate an association between maternal plasma taurine concentrations during pregnancy and offspring birth weight in humans. One previous study linked lower dietary taurine intake during pregnancy with lower offspring birth weight [54], consistent with findings from animal studies [55,56]. A recent study investigated associations between serum taurine concentrations and offspring anthropometrics at delivery and found no significant associations [57]. However, taurine concentrations differ significantly between serum and plasma due to leakage from platelets and granulocytes, both of which contain substantial amounts of taurine. This limits the interpretability of studies measuring serum taurine and hinders comparisons with plasma taurine concentrations [30].

Our results suggest that taurine is linked to birth weight, and an adequate taurine intake during pregnancy might therefore be important for fetal growth. In adults, taurine is primarily synthesized from methionine and cysteine in the liver, although several other organs, including the brain, kidneys, adipose tissue, and mammary glands, can also contribute to its production. While the capacity for taurine synthesis in the human placenta remains unknown, mRNA expression of the necessary enzymes has been detected [19], and the placenta has been shown to release taurine into both the maternal and fetal circulations [53]. Notably, the placenta lacks the enzymes required for the conversion of methionine to cysteine [18], suggesting that placental taurine synthesis is dependent on maternally supplied cysteine. 

Taurine appears to play a critical role in fetal development. Studies in cats have demonstrated that severely deficient taurine plasma concentrations can result in stillbirth, low birth weight, and neurological defects [14]. Dietary intake of meat and seafood is the primary source of taurine for most individuals [13]. Given the increasing prevalence of vegetarian and vegan diets, further investigation into taurine metabolism during pregnancy is particularly important. 

### 4.5. Strengths and Weaknesses

To our knowledge, this is the first study to investigate the effects of PA during pregnancy on plasma concentrations of SCAAs in humans. A primary strength of this study is its randomized controlled trial design and the use of repeated measurements of SCAAs, allowing us to track metabolic changes throughout pregnancy. Furthermore, the consistent timing of plasma sample collection during pregnancy is a strength, as maternal metabolic changes and fetal demand can alter AA plasma concentrations throughout gestation. However, several limitations must be considered. 

The lack of dietary records for participants is a notable weakness, as a vegetarian or vegan diet can significantly influence plasma concentrations of SCAAs [58,59,60]. More broadly, plasma concentrations of methionine and cysteine/cystine are highly sensitive to the intake of protein-rich foods, such as meat, fish, shellfish, and eggs [13]. While we cannot account for individual dietary variations, the randomized design of the FitMum study suggests that baseline dietary habits were likely balanced across the three study groups, mitigating the risk of systemic dietary bias. 

Maternal plasma samples were collected in a non-fasting state. Most AAs increase in serum after a meal [61], and one study found that fasting can significantly decrease methionine plasma concentrations with no change in cysteine [62], while another found no effect of fasting on both methionine, cysteine, and taurine plasma concentrations [63]. Because the non-fasting state was consistent across all study groups, it is unlikely to have introduced systematic bias in the comparative analysis between groups. However, the resulting variation may have introduced random measurement error, which could potentially attenuate the strength of the observed associations with neonatal outcomes. In addition, we were unable to adjust for delivery-specific variables that could influence the plasma concentrations of SCAAs, such as labor duration, mode of delivery, intravenous fluid administration, recent food intake, and interval between delivery and blood collection.

While this study provides a novel look at plasma concentrations of SCAAs in pregnancy, the inclusion of plasma homocysteine measurements in future studies would offer a complementary perspective on metabolism and homeostasis of SCAAs. Due to its role as the central intermediate between methionine and cysteine, quantifying homocysteine would allow for a more detailed understanding of the observed changes in the metabolic regulation of SCAAs, further enhancing the insights gained here regarding the effects of PA on maternal-fetal nutrient sulfur amino acid metabolism.

Additionally, our measurement of tCys as cystine does not allow us to distinguish between the reduced and oxidized forms in vivo. Given that the redox balance is influenced by pregnancy and exercise, the inability to quantify the specific ratio of cysteine to cystine is a limitation, as the biological activity of these molecules depends on their oxidation state.

Another limitation is that maternal plasma AA concentrations were measured in EDTA plasma, while umbilical cord plasma concentrations were measured in CPD plasma. However, comparisons of CPD and EDTA plasma in a subset of maternal samples revealed no difference in methionine or tCys concentrations. We observed a small difference in taurine plasma concentrations between CPD and EDTA plasma, with EDTA plasma showing on average 1.15 times higher concentrations compared to CPD plasma (Appendix A, Table A1). To address this, we attempted to adjust umbilical cord plasma taurine concentrations by multiplying by 1.15, which did not alter the results.

Regarding generalizability, it is note-worthy that the participants in the FitMum study were a selected population of healthy, previously inactive, and highly educated women. Therefore, these findings may not be fully representative of the general pregnant population, and larger differences might be observed in more heterogenous groups.

Lastly, as this was a secondary analysis of the FitMum RCT, several outcomes were explored without a formal correction for multiple comparisons. Consequently, these findings should be interpreted as exploratory, and the observed associations warrant further validation in future prospective studies.

## 5. Conclusions

Methionine and taurine concentrations decreased during pregnancy, while total cysteine concentrations increased, suggesting that metabolic changes during pregnancy alter maternal plasma concentrations of SCAAs. Increased PA during pregnancy increased maternal plasma concentrations of methionine and total cysteine, but further research is needed to confirm this. PA does not appear to affect taurine plasma concentrations, but taurine seemed to play an important role in fetal growth, as higher taurine concentrations were associated with increased offspring birth weight.

## Figures and Tables

**Table 1:** Maternal and neonatal characteristics. Presented as mean ± standard deviation or as median with interquartile range for skewed data.

**Maternal Characteristics**	***n* = 214**	**CON (** ***n* = 44)**	**EXE (** ***n* = 86)**	**MOT (** ***n* = 84)**
Age (years)	31.4 ± 4.3	31.8 ± 4.4	31.0 ± 4.3	31.7 ± 4.2
Pre-pregnancy BMI ^a^ (kg/m^2^)	24.1 (21.9–28.7)	23.6 (21.3–26.9)	25.1 (21.6–29.6)	24.2 (22.4–28.9)
GA at inclusion (weeks)	12.6 (9.3–13.7)	12.9 (9.6–13.9)	12.6 (9.3–13.7)	12.7 (9.4–13.8)
Nullipara	82 (38%)	16 (36%)	40 (47%)	26 (31%)
Educational level ^b^				
Further education ≥ 3 years	170 (79%)	32 (73%)	72 (84%)	66 (79%)
MVPA ^c^ (min/week)	27.2 ± 21.4	25.5 ± 14.9	27.1 ± 20.5	29.1 ± 26.8
**Neonatal Characteristics**	***n* = 177**	**CON (** ***n* = 34)**	**EXE (** ***n* = 74)**	**MOT (*n* = 69)**
Birth weight (g)	3634 ± 525	3515 ± 589	3738 ± 489	3580 ± 516
Placental weight (g) ^d^	681 ± 142	656 ± 172	691 ± 129	683 ± 139
BW/PW ^d^	5.4 ± 0.9	5.5 ± 0.7	5.4 ± 1.1	5.4 ± 0.8
Birth length (cm)	51.9 ± 2.7	51.9 ± 2.7	52.1 ± 2.8	51.6 ± 2.7
Head circumference (cm)	35.3 ± 1.6	34.8 ± 2.1	35.4 ±1.3	35.3 ±1.5
GA at delivery (weeks)	40.4 (39.6–41.1)	40.2 (38.8–41.3)	40.6 (39.9–41.3)	40.0 (39.3–40.9)
Sex				
Male	84 (47%)	13 (38%)	42 (57%)	29 (42%)
Female	93 (53%)	21 (62%)	32 (43%)	40 (58%)

BMI, Body mass index; GA, gestational age; BW/PW, birth weight/placental weight ratio; MVPA, moderate-to-vigorous intensity physical activity; EXE, Structured supervised exercise training; MOT, Motivational counseling on physical activity; CON, Standard care. ^a^ Pre-pregnancy BMI; *n* = 213. ^b^ Further education ≥ 3 years corresponds to a university degree (bachelor’s or master’s level). ^c^ Weekly mean from randomization to delivery. ^d^ For placental weight, and BW/PW; *n* = 172.

**Table 2:** Estimated mean concentrations of sulfur-containing amino acids in maternal plasma at GA <15+0, 28+0-6, 34+0-6, and at delivery with 95% confidence intervals (CI). Differences between visits were analyzed using linear mixed models. Median concentrations in umbilical cord plasma with interquartile range (IQR). Differences between maternal plasma concentrations at delivery and umbilical cord plasma concentrations were tested with paired *t*-tests for methionine and tCys and with Wilcoxon signed-rank test for taurine.

	GA <15+0 * Mean [95% CI]	GA 28+0-6 Mean [95% CI] *p*-Value	GA 34+0-6 Mean [95% CI] *p*-Value	Delivery Mean [95% CI] *p*-Value	Umbilical Median (IQR)	Umbilical vs. Maternal *p*-Value
**Methionine (** **μ** **mol/L)**	21.4 [20.5–22.3]	21.9 [21.0; 22.9] 0.399	22.7 [21.8; 23.7] 0.030	19.2 [18.1; 20.2] <0.001	6.8 (3.9–10.6)	<0.001
**tCys (** **μ** **mol/L)**	36.1 [34.3; 37.9]	32.3 [30.3; 34.2] <0.001	34.1 [32.1; 36.1] 0.095	46.0 [44.0; 48.1] <0.001	6.5 (3.6–9.7)	<0.001
**Taurine (** **μ** **mol/L)**	65.4 [62.0; 68.8]	47.7 [44.0; 51.4] <0.001	45.3 [41.5; 49.1] <0.001	41.6 [37.6; 45.5] <0.001	35.1 (17.7–52.6)	<0.001

GA, Gestational age in weeks + days; tCys, total cysteine; * reference values.

**Table 3:** Results from baseline constrained linear mixed models with estimated mean values of methionine, total cysteine, and taurine concentrations in CON, EXE, and MOT at gestational age week 28+0-6, 34+0-6, and at delivery. Presented as estimated means with 95% confidence intervals (CI) and *p*-values. All analyses adjusted for analysis batch.

	Week 28+0-6		Week 34+0-6		Delivery	
	Estimate [95% CI]	*p*-Value	Estimate [95% CI]	*p*-Value	Estimate [95% CI]	*p*-Value
**Methionine (µmol/L)**						
CON *	18.98 [16.61; 21.35]		21.83 [19.45; 24.21]		18.90 [16.69; 21.10]	
EXE	21.93 [19.25; 24.61]	**0.031**	22.81 [20.12; 25.50]	0.477	19.17 [16.72; 21.62]	0.829
MOT	22.77 [20.05; 25.49]	**0.006**	22.40 [19.66; 25.13]	0.687	18.52 [16.05; 20.99]	0.776
**tCys (µmol/L)**						
CON *	27.98 [24.78; 31.19]		31.46 [27.78; 35.13]		42.97 [37.17; 48.77]	
EXE	30.23 [27.05; 33.41]	0.167	32.73 [28.90; 36.55]	0.516	45.52 [38.77; 52.27]	0.460
MOT	31.92 [28.69; 35.15]	**0.017**	32.23 [28.33; 36.13]	0.698	44.01 [37.22; 50.80]	0.765
**Taurine (µmol/L)**						
CON *	45.67 [39.58; 51.76]		44.50 [38.24; 50.76]		41.23 [26.84; 55.63]	
EXE	48.72 [42.56; 54.88]	0.332	43.59 [37.03; 50.16]	0.787	36.86 [19.79; 53.93]	0.616
MOT	45.03 [38.78; 51.28]	0.841	44.75 [38.08; 51.42]	0.942	44.32 [27.15; 61.49]	0.725

* Reference group. CON, Standard care; EXE, Structured supervised exercise training; MOT, Motivational counseling on physical activity; tCys, total cysteine. Significant *p*-values are highlighted in bold.

**Table 4:** Results from linear regression analyses with estimated mean differences of methionine, total cysteine, and taurine concentrations in maternal plasma at delivery minus umbilical cord plasma (MUV-difference) in CON, EXE, and MOT. Presented as estimated mean differences with 95% confidence intervals (CI) and *p*-values. Adjusted for analysis batch and hemoglobin plasma concentration.

	MUV-Difference	
	Estimate [95% CI]	*p*-Value
**Methionine (µmol/L)**		
CON *	10.20 [6.33; 14.07]	
EXE	11.71 [7.44; 15.99]	0.484
MOT	13.12 [8.76; 17.49]	0.187
**tCys (µmol/L)**		
CON *	26.50 [17.83; 35.17]	
EXE	30.87 [21.40; 40.33]	0.361
MOT	29.09 [19.15; 39.03]	0.605
**Taurine (µmol/L)**		
CON *	0.35 [−18.16; 18.86]	
EXE	5.29 [−15.15; 25.74]	0.632
MOT	11.24 [−9.84; 32.33]	0.307

* Reference group. MUV-difference, maternal–umbilical venous difference; CON, Standard care; EXE, Structured supervised exercise training; MOT, Motivational counseling on physical activity; tCys, total cysteine.

**Table 5:** Effect modification of MVPA and sedentary time from randomization to delivery on associations between gestational age and methionine, total cysteine, and taurine plasma concentrations. Presented as estimated change in methionine, total cysteine, and taurine plasma concentrations, respectively, per 1 unit increase in exposure with 95% confidence intervals (CI). Adjusted for maternal age, pre-pregnancy body mass index, and analysis batch.

	MVPA (min/Day)			Sedentary Time (h/Day)		
	Slope [95% CI]	SE	*p*-Value	Slope [95% CI]	SE	*p*-Value
**Methionine**						
GA (weeks) PA measure GA:PA measure	−0.05 [−0.12; 0.02]	0.035	0.156	−0.07 [−0.53; 0.39]	0.234	0.777
	−0.03 [−0.12; 0.07]	0.047	0.587	−3.1 × 10^−3^ [−0.96; 0.97]	0.492	0.995
	6.5 × 10^−4^ [−1.9 × 10^−3^; 3.2 × 10^−3^]	0.001	0.616	2.1 × 10^−3^ [−0.03; 0.03]	0.017	0.902
**tCys**						
GA (weeks) PA measure GA:PA measure	−0.20 [−0.37; −0.03]	0.087	**0.024**	−1.09 [−2.15; −0.03]	0.540	**0.044**
	−0.13 [−0.33; −0.08]	0.106	0.235	−0.68 [−2.92; 1.56]	1.141	0.554
	5.0 × 10^−3^ [1.2 × 10^−3^; 0.01]	0.003	0.118	0.07 [−0.01; 0.15]	0.041	0.072
**Taurine**						
GA (weeks) PA measure GA:PA measure	−0.82 [−1.09; −0.55]	0.141	**<0.001**	−4.36 [−8.36; −0.37]	2.035	**0.032**
	−0.32 [−0.65; 0.02]	0.171	0.065	−10.18 [−20.09; −0.27]	5.046	**0.044**
	4.1× 10^−3^ [−4.3 × 10^−3^; 0.01]	0.004	0.338	0.27 [−0.03; 0.57]	0.154	0.078

GA, Gestational age; MVPA, moderate-to-vigorous intensity physical activity; GA:PA measure, change in MVPA or sedentary time when GA increases; tCys, total cysteine. Significant *p*-values are highlighted in bold.

**Table 6:** Associations between minutes of moderate-to-vigorous intensity physical activity and sedentary time, respectively, and differences in maternal and umbilical cord plasma concentrations of methionine, total cysteine, and taurine at delivery with 95% confidence intervals (CI). Adjusted for maternal age, and pre-pregnancy body mass index, analysis batch, and umbilical cord plasma hemoglobin concentration.

	MUV-Difference	
	Estimate [95% CI]	*p*-Value
**MVPA (min/day)**		
Methionine (µmol/L)	−0.12 [−0.22; −0.02]	**0.025**
tCys (µmol/L)	−0.08 [−0.30; 0.14]	0.474
Taurine (µmol/L)	−0.43 [−0.95; 0.08]	0.099
**Sedentary time (hours/day)**		
Methionine (µmol/L)	−1.31 [−3.45; 0.84]	0.231
tCys (µmol/L)	2.55 [−2.50; 7.60]	0.317
Taurine (µmol/L)	−5.69 [−16.88; 5.50]	0.315

MUV-difference, maternal–umbilical venous difference; MVPA, moderate-to-vigorous intensity physical activity; tCys, total cysteine. Significant *p*-values are highlighted in bold.

**Table 7:** Associations between maternal (Model 1 and Model 2) or umbilical cord (Model 3) methionine, total cysteine, and taurine plasma concentrations during all time points in pregnancy and placental weight, birth weight, birth weight/placental weight ratio, birth length, and offspring head circumference.

	Model 1 (*n* = 196)		Model 2 (*n* = 196)		Model 3 (*n* = 97)	
	Estimate	*p*-Value	Estimate	*p*-Value	Estimate	*p*-Value
**Methionine**						
Placental weight (g)	0.42 [−2.56; 3.40]	0.782	0.12 [−2.97; 3.21]	0.939	5.19 [−1.39; 11.77]	0.120
Birth weight (g)	4.89 [−4.39; 14.17]	0.302	8.89 [−1.60; 19.37]	0.098	−0.13 [−24.58; 24.33]	0.992
BW/PW	8.3 × 10^−3^ [−0.02; 0.04]	0.567	0.01 [−0.02; 0.05]	0.369	−0.03 [−0.09; 0.04]	0.453
Birth length	9.2 × 10^−3^ [−0.03; 0.05]	0.670	0.03 [−0.01; 0.08]	0.174	0.05 [−0.04; 0.15]	0.288
Head circumference	2.3 × 10^−3^ [−0.03; 0.04]	0.892	0.02 [−0.02; 0.05]	0.374	−3.9 × 10^−3^ [−0.08; 0.07]	0.918
**tCys**						
Placental weight (g)	−0.47 [−2.27; 1.33]	0.610	−0.93 [−2.89; 1.03]	0.354	3.41 [−0.87; 7.70]	0.116
Birth weight (g)	−2.23 [−7.94; 3.48]	0.444	−2.49 [−8.50; 3.51]	0.416	11.85 [−4.14; 27.83]	0.142
BW/PW	3.0 × 10^−4^ [−0.02; 0.02]	0.975	4.1 × 10^−3^ [−0.02; 0.02]	0.685	−1.7 × 10^−3^ [−0.05; 0.04]	0.941
Birth length	−0.01 [−0.04; 9.3 × 10^−3^]	0.250	−0.02 [−0.04; 8.1 × 10^−3^]	0.198	0.04 [−0.03; 0.11]	0.243
Head circumference	−0.03 [−0.04; −0.01]	**0.001**	−0.03 [−0.04; −0.01]	**<0.001**	0.04 [−0.01; 0.09]	0.146
**Taurine**						
Placental weight (g)	−0.29 [−0.82; 0.24]	0.290	−0.70 [−1.54; 0.14]	0.104	0.28 [−0.95; 1.51]	0.647
Birth weight (g)	2.11 [−0.04; 4.27]	0.055	3.25 [0.26; 6.25]	**0.034**	−1.55 [−5.97; 2.87]	0.485
BW/PW	5.9 × 10^−3^ [4.1 × 10^−4^; 0.01]	**0.036**	0.01 [2.7 × 10^−3^; 0.02]	**0.010**	−3.2 × 10^−3^ [−0.02; 8.9 × 10^−3^]	0.600
Birth length	7.2 × 10^−3^ [−1.9 × 10^−3^; 0.02]	0.121	6.4 × 10^−3^ [−4.5 × 10^−3^; 0.02]	0.248	5.9 × 10^−3^ [−0.01; 0.02]	0.495
Head circumference	5.5 × 10^−3^ [−1.4 × 10^−3^; 0.01]	0.119	5.7 × 10^−3^ [−3.2 × 10^−3^; 0.01]	0.210	−5.8 × 10^−3^ [−0.02; 7.8 × 10^−3^]	0.397

Model 1: Analyses with maternal plasma concentrations at all time points in pregnancy only adjusted for analysis batch. Model 2: Analyses with maternal plasma concentrations at all time points in pregnancy adjusted for maternal age, gestational age, offspring sex, and maternal pre-pregnancy BMI, and analysis batch. Model 3: Analyses with umbilical cord plasma adjusted for maternal age, gestational age, offspring sex, and maternal pre-pregnancy BMI, analysis batch, and hemoglobin concentration. BW/PW, birth weight/placental weight ratio; tCys, total cysteine. Significant *p*-values are highlighted in bold.

## Data Availability

The data presented in this study are available on request from the corresponding author due to confidentiality. Individual participant data will be transferred according to the Data Protection Act, when approval from the Danish Data Protection Agency is obtained, and a Standard Contractual Clause is completed to ensure the legal basis of the transfer.

## Abbreviations

The following abbreviations are used in this manuscript:

AA	Amino acid
BMI	Body mass index
BW/PW	Birth weight/placental weight ratio
CON	Standard care
EXE	Structured supervised exercise training
GA	Gestational age
MOT	Motivational counseling on physical activity
MRM	Multiple-reaction monitoring
MVPA	Moderate-to-vigorous physical activity
PA	Physical activity
QC	Quality control
SCAAs	Sulphur-containing amino acids
tCys	Total cysteine

## Appendix A

**Table A1:** Concentrations of methionine, total cysteine (tCys), and taurine in EDTA and CPD plasma, respectively, presented as mean values ± standard deviation (*n* = 10). Difference between EDTA and CPD was tested with a paired *t*-test.

	EDTA	CPD	*p*-Values
Methionine	18.2 ± 6.6	17.4 ± 5.4	0.222
tCys	31.3 ± 8.9	33.5 ± 7.6	0.104
Taurine	28.4 ± 7.1	24.6 ± 4.4	0.012
